# Progressive encephalomyelitis with rigidity and myoclonus (PERM) associated with anti-glycine receptor antibodies and urothelial carcinoma: a case report

**DOI:** 10.1186/s13256-023-04059-w

**Published:** 2023-08-03

**Authors:** Amanuel Hassen Ali, Anna Benterud, Trygve Holmøy, Aija Zuleron Myro

**Affiliations:** 1 Department of Neurology, Akershus University Hospital, Lørenskog, Norway; 2 Institute of Clinical Medicine, University of Oslo, Oslo, Norway

**Keywords:** PERM, SPSD, GlyR-antibody, Urothelial carcinoma, Myoclonus, Trismus, Rigidity

## Abstract

**Background:**

Progressive encephalomyelitis with rigidity and myoclonus (PERM) is a rare neurological condition with paraneoplastic etiology in about 20% of cases, usually presenting before or shortly after the oncological diagnosis is established. PERM associated with anti-glycine receptor antibodies is not previously reported in a patient with bladder cancer.

**Case presentation:**

A 72-years-old Caucasian male was admitted with acute onset of dysarthria, dysphagia and trismus three years after initial surgical treatment for bladder cancer. The condition was initially diagnosed as tetanus and treated accordingly, but the diagnosis was reconsidered because of progression despite adequate treatment. Diagnostic workup on readmission revealed lung and paraaortal metastases from bladder cancer and anti-glycine receptor (anti-GlyR) antibodies both in the cerebrospinal fluid and in serum, which supplemented with the clinical presentation led to the diagnosis of PERM, presumably related to bladder cancer. The patient showed improvement and stabilization after treatment with intravenous immunoglobulin and chemotherapy against metastatic bladder cancer.

**Conclusion:**

To the best of our knowledge, this is the first reported case of anti-GlyR antibody positive PERM related to urothelial carcinoma. The symptoms mimicked tetanus, and responded to chemotherapy and immunotherapy.

## Background

Progressive encephalomyelitis with rigidity and myoclonus (PERM) is part of the spectrum of stiff-person syndrome disorders (SPSD) and presents as limb and truncal rigidity, painful muscle spasms, brainstem signs and hyperreflexia [1]. PERM is more severe and more often fatal than the classical form of SPSD and affects men more often than women [2]. The presentation can be acute, subacute or exacerbation on a chronic course [3]. The most frequent antibodies associated with PERM include antibodies targeting glutamic acid decarboxylase (GAD), glycine receptors (GlyR), *N*-methyl-d-aspartate receptors (NMDAR), and dipeptidyl-peptidase-like protein 6 (DPPX) [3, 4]. If untreated, PERM tends to progress to death within 2–3 years, in some cases more rapidly.

About 20% of PERM cases are paraneoplastic, and association with small cell lung cancer, lymphomas, melanomas, breast cancer and thymomas is well documented [5–8]. We describe the first case of PERM associated with anti-glycine receptor antibodies and urothelial carcinoma. Patients with PERM associated with anti-GlyR antibody tend to respond better to treatment than the classical form of SPSD [3, 4, 9].

## Case presentation

A 72-years-old Caucasian male was admitted to neurological department because of subacute onset of painful tongue weakness and dysphagia starting three days before admission. The patient was operated three years back with transurethral resection of papillary urothelial cancer, but did not show up to scheduled control and declined adjuvant cytostatic treatment. He was re-operated with cystectomy two years and three months after the first operation because of recurrence of the urothelial cancer with local lymph node metastases. The patient declined further cytostatic treatment. He was otherwise healthy, and no family history of neurological diseases was noted.

**Fig. 1:**
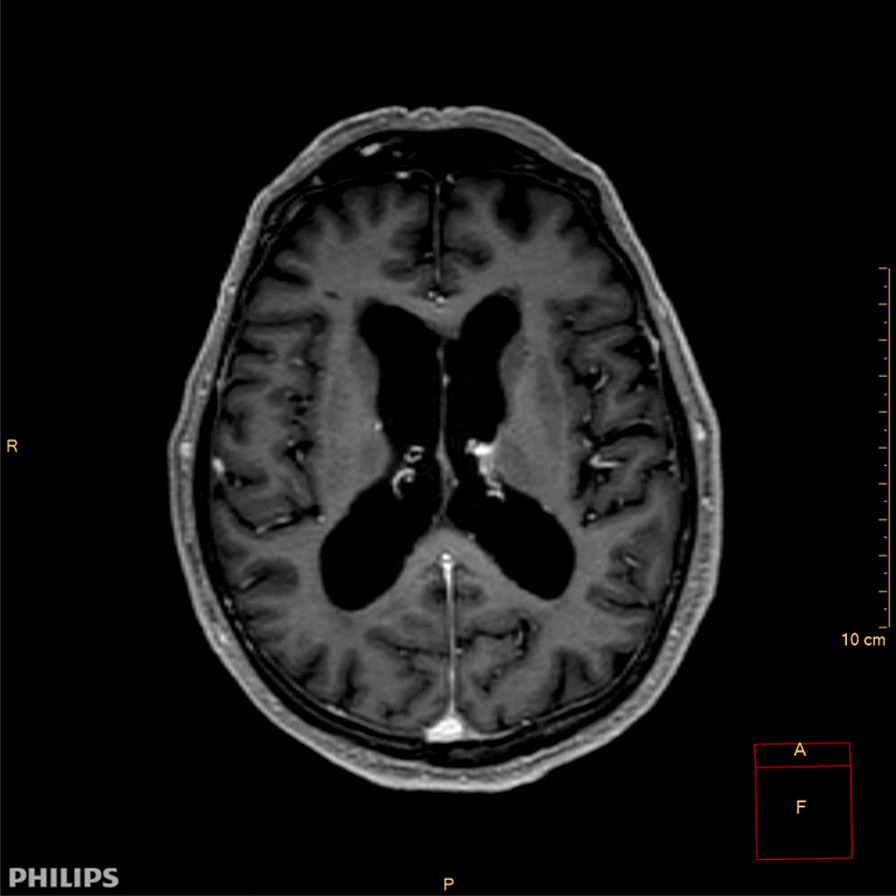
Transverse longitudinal relaxation time (T1) gadolinium enhanced magnetic resonance imaging (MRI) of the brain showing mild central atrophy, otherwise normal. (CM: centimeter; R: right; P: posterior; A: focus; cm: centimeter)

Three days after debut of weakness of the tongue, the patient developed increasing dysphagia, dysarthria, lockjaw and painful face spasms. Clinical examination at the first presentation to department of neurology revealed moderate dysarthria, dysphagia, dysconjugated eye movements and inverted plantar reflex on the left side. Magnetic resonance imaging (MRI) of the brain showed mild central atrophy, but was otherwise normal (Fig. 1). Analyses of cerebrospinal fluid was unremarkable except for mild pleocytosis with 8 mononuclear cells per µL. Anti-NMDA, anti-AMPA receptors 1 and 2, GABA B receptors 1 and 2, anti-LGI1, anti-CASPR2 and anti-DPPX IgG in CSF were all negative. Paraneoplastic antibodies in serum including Anti-GAD 65 and anti-amphipyhsin were negative. Anti-GlyR antibody was not analyzed at that time.

Based on clinical presentation dominated by lockjaw and stimulus sensitive painful spasms, tetanus was suspected, and the patient was treated with intravenous Penicillin, tetanus immunoglobulin and symptomatic treatment with diazepam. The patient experienced significant improvement of painful spasms and swallowing difficulties, and he was discharged 20 days after admission.

One month later the patient was re-admitted because of deterioration of his general medical condition and ambulation, with increasing painful spasms in the extremities aggravated by noises and tactile stimulation, lockjaw, swallowing difficulties, slurred speech and diplopia. The symptoms were still attributed to tetanus and the patient was treated with diazepam, baclofen and onabotulinum toxin along with intensive physiotherapy at the department of neurological rehabilitation for about one month but did not improve. No further investigation was carried out at that time.

**Fig. 2:**
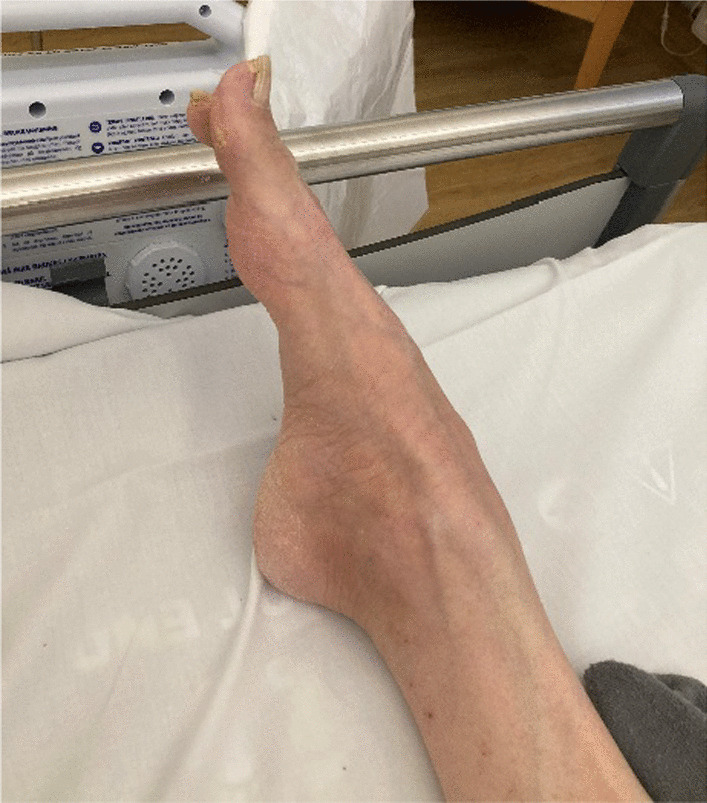
Picture of the right foot of the patient showing pes equinus deformity

The patient was re-admitted to department of neurology 3 weeks after discharge from the rehabilitation center because of sudden bouts of stiffness in the whole body accompanied by cyanosis around the lips and brief episodes of unconsciousness responding to diazepam. He also described hallucinations and delusion. Neurological examination showed myoclonic jerks, painful spasms triggered by passive movements of the extremities and bilateral pes equinus (Fig. 2). Electroencephalography on three different occasions did not show definite epileptiform activity. Tetanus was then considered unlikely because of progression despite adequate treatment.

Immunofluorescence assay for paraneoplastic antibodies analyzed at the Clinical immunology laboratory in Groß Grönau, Germany, was strongly positive for anti-GlyR IgG both in serum (1:3200) and CSF (1:100). Routine examination of CSF including isoelectric focusing of IgG, and also immunofluorescence assays for IgG antibodies against amphiphysin, GAD 65, titin, NMDAR, AMPAR1/2, DPPX, GABRB1, CASPR2 and LGI1, were negative.

**Fig. 3:**
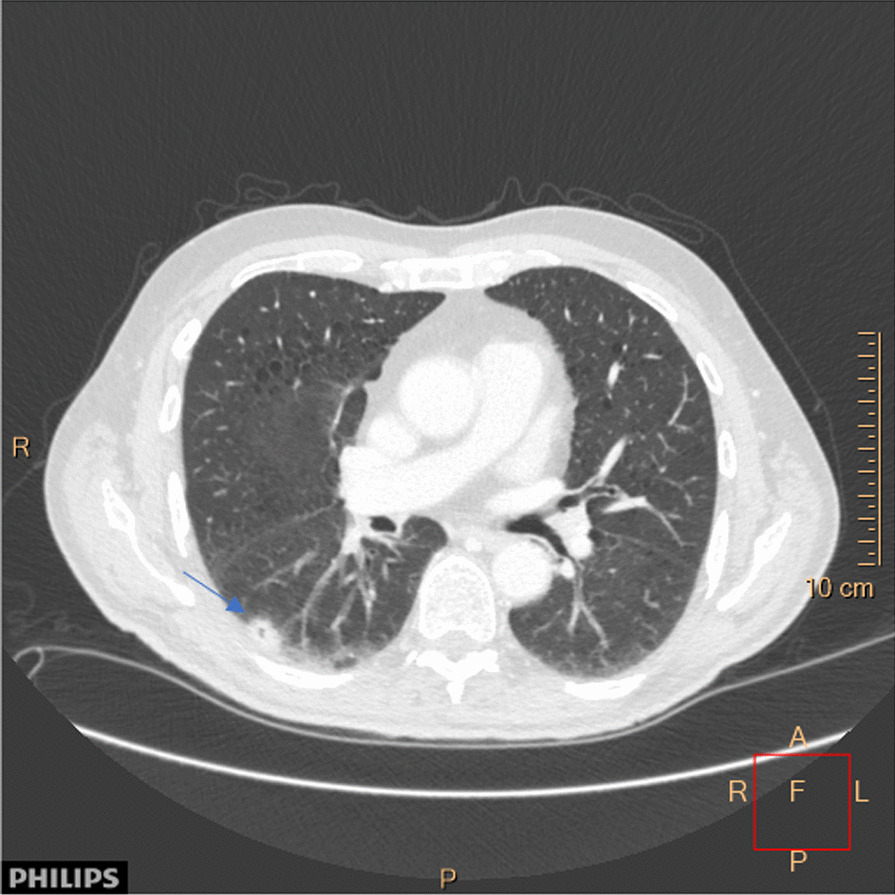
Computed tomography (CT) of the thorax axial view taken before the initiation of chemotherapy showing nodulus (blue arrow) in the lower lobe of right lung. (A: anterior; R: right; L: left; P: posterior, F: focus; cm: centimeter)

Electromyography showed simultaneous activation of motor units in agonist and antagonist muscles both in the upper and lower extremities. CT thorax showed nodulus in the lower lobe of the right lung (Fig. 3) and CT of the abdomen showed enlarged paraaortal lymph nodes. Biopsy from the lung lesion was consistent with metastases from urothelial carcinoma.

**Fig. 4:**
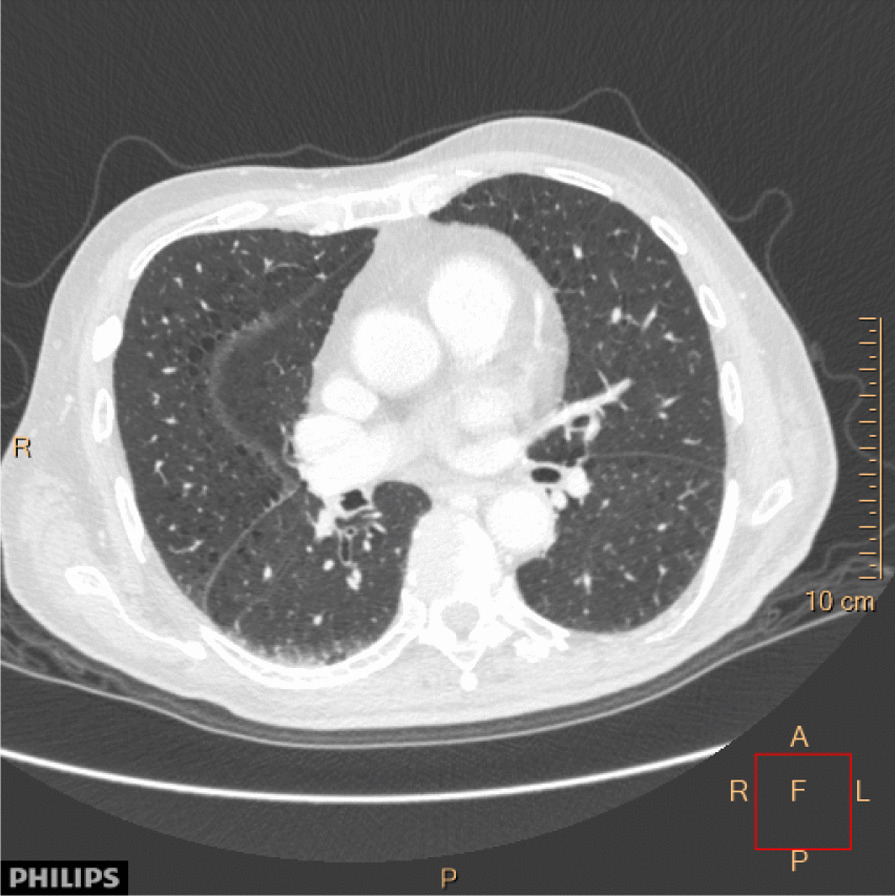
Computed tomography (CT) of the thorax axial view taken 2 months after the initiation of chemotherapy showing significant regression of the lesion in the lower lobe of the right lung. (A: anterior; R: right; L: left; P: posterior, F: focus; cm: centimeter)

A diagnosis of PERM associated with anti-GlyR antibody was made. The patient was initiated on intravenous immunoglobulin 25 g given daily for 5 consecutive days every fourth week, and at the same time referred to oncologist for further oncological treatment. He received total of 6 cycles with immunoglobulin. Cytostatic treatment was initiated after the second course of immunoglobulin therapy which was  three months after onset of neurological symptoms. The patient completed three of four scheduled courses of cytostatic treatment with gemcitabine/cisplatin given with three weeks interval (day 1: intravenous gemcitabin 1360 mg, day 2: cisplatin 95 mg, day 8: gemcitabine 1360 mg). Control CT scan 2 months after initiation of cytostatic treatment showed reduction of the lung lesion (Fig. 4) and paraaortal lymph nodes. The fourth course of chemotherapy was not given as the patient declined further cytostatic treatment. The patient experienced significant improvement regarding painful spasms and daily function, and became able to sit in wheelchair and perform many daily activities with some help. Treatment with intravenous immunoglobulin was discontinued after 6 months due to stabilization of neurological function though retaining significant neurological disability.

At follow up 8 months after the last chemotherapy the patient exhibited significant weight loss, reduced appetite, diffuse pain in different parts of the body and blood chemistry showed high level of human chorionic gonadotropin. In parallel with the worsening of the oncological condition, the patient experienced worsening of symptoms relating to PERM, especially rigidity and spasms in the extremities. Imaging studies were considered, but the patient did not want further investigation and treatment. He died about one year after diagnosis of PERM.

## Discussion

The diagnosis of PERM is often challenging. The clinical presentation of PERM can include trismus and thus mimic tetanus as in our patient. The cardinal symptoms of PERM, including muscle rigidity, painful spasms, dysphagia and myoclonus occur also in patients with tetanus, though the degree and localization may vary. Voluntary movements and tactile or emotional stimulation may trigger spasms in both PERM and tetanus. There is often a preceding history of trauma and spasms of the masseter muscle is often the first symptom in tetanus [10, 11].

Trismus as the initial presentation of PERM is previously described in an antibody negative patient with underling ovarian teratoma [12]. As in our case the patient was initially treated for tetanus before PERM was diagnosed. Though tetanus is rare in Norway, the condition was suspected in our patient based on the clinical presentation and finding of bad oral hygiene which was assumed to be entry route. Lack of sustainable improvement despite adequate treatment prompted reassessment of the diagnosis.

Our patient presented with subacute onset of brainstem and cerebral symptoms consistent with an inflammatory etiology. In Norway lumbar puncture is routinely performed in such patients to exclude possible infectious etiologies including borreliosis, listeriosis and viruses. CSF analysis is more sensitive than serum analysis for certain antibodies such as anti-NMDA receptor IgG, anti-GAD65 IgG, and anti-GFAP IgG, whereas serum is more sensitive for other antibodies such as anti-LGI1 IgG and anti-Caspr2 IgG [13].

In adults, glycine receptors are expressed predominantly in the spinal cord and brainstem where, together with GABA_A_ receptors, they mediate fast inhibitory neurotransmission [14]. GlyR antibodies are associated with wide variety of neurological disorders including PERM, limbic encephalitis, epileptic encephalopathy, brainstem features and demyelinating optic neuropathies [15]. The mechanism of GlyR antibody action is not clearly understood, but suppression of GlyR function is assumed to induce excessive response to sensory inputs, such as those from muscle spindles, light and sound stimuli [16]. In line with this, purified IgG from patients with GlyR antibodies were recently shown to inhibit synaptic currents in cultured motor neurons, supporting that the autoantibodies are pathogenic in PERM [17]. There are reports of GlyR antibodies related PERM associated with small cell lung cancer, lymphomas, melanomas, breast cancer and thymomas [5–7]. To the best of our knowledge, this is the first case of PERM with GlyR antibodies associated with bladder cancer.

In our patient cancer preceded PERM. Moreover, he was treated with both IVIG and chemotherapy, and it is therefore possible that improvement of PERM was due to IVIG rather than to cancer treatment. Thus, we cannot exclude that the relationship between bladder cancer and PERM was coincidental rather than causal. The onset of PERM did however coincide with discovery of lung and paraaortal metastases, supporting a causal relationship.

## Conclusion

This case report highlights the importance of having in mind the possibility of paraneoplastic conditions in patients with long standing progressive cancer, and suggests that investigations for bladder cancer should be considered in the diagnostic work up of PERM.

## Data Availability

Data sharing is not applicable to this article as no datasets were generated or analyzed during the current study.

## Abbreviations

PERM: Progressive encephalomyelitis with rigidity and myoclonus
SPSD: Spectrum of stiff-person syndrome
anti-GlyR: Anti-glycine receptor
GAD: Glutamic acid decarboxylase
NMDAR: *N*-Methyl-d-aspartate receptors
DPPX: Dipeptidyl-peptidase-like protein
GABRB1: Gamma-aminobutyric acid type A receptor subunit B1
CASPR2: Contactin-associated protein-like 2
LGI1: Leucine-rich, glioma inactivated 1
MRI: Magnetic resonance imaging
anti-AMPA: Anti-α-amino-3-hydroxy-5-methyl-4-isoxazolepropionic acid
GABA: Gamma-aminobutyric acid

## References

[1] Hutchinson M, Walters P, McHugh J (2008). Progressive encephalomyelitis, rigidity, and myoclonus: a novel glycine receptor antibody. Neurology.

[2] Stern WM, Howard R, Chalmers RM (2014). Glycine receptor antibody mediated progressive encephalomyelitis with rigidity and myoclonus (PERM): a rare but treatable neurological syndrome. Pract Neurol.

[3] Dalakas MC, Fujii M, Li M (2001). High-dose intravenous immune globulin for stiff-person syndrome. N Engl J Med.

[4] Crisp SJ, Balint B, Vincent A (2017). Redefining progressive encephalomyelitis with rigidity and myoclonus after the discovery of antibodies to glycine receptors. Curr Opin Neurol.

[5] Kyskan K, Chapman K, Mattman A (2013). Antiglycine receptor antibody and encephalomyelitis with rigidity and myclonus (PERM) related to small cell lung cancer. BMJ Case Rep.

[6] Su Y, Cui L, Zhu M (2020). Progressive encephalomyelitis with rigidity and myoclonus with Thymoma: a case report and Literature review. Front Neurol.

[7] Borellini L, Lanfranconi S, Bonato S (2017). Progressive encephalomyelitis with rigidity and myoclonus associated with anti-GlyR antibodies and Hodgkins’s lymphoma: a case report. Front Neurol.

[8] Baizabal-Carvallo JF, Jankovic J (2015). Stiff-person syndrome: insights into a complex autoimmune disorder. J Neurol Neurosurg Psychiatry.

[9] Chang A, Lin KY, Chuang KJ (2021). Progressive encephalomyelitis with rigidity: a Taiwanese case and review of literature. Clin Neurol Neurosurg.

[10] Giannini L, Maccari A, Chiesa V (2016). Trismus, the first symptom in a challenging diagnosis of Tetanus. BMG Case Rep.

[11] Olsen BC, Stubhaug TT, Berild JD (2019). A woman in her fifties with trismus and muscle spasms. Tidsskr Nor Legeforen.

[12] Blomme L, Van De Velde K (2019). Trismus as a presenting symptom in a case of progressive encephalopathy with rigidity and myoclonus. Case Rep Neurol.

[13] Abbatemarco JR, Yan C, Kunchok A (2021). Antibody-mediated autoimmune encephalitis: a practical approach. Clevel Clin J Med.

[14] Lynch JW (2009). Native glycine receptor subtypes and their physiological roles. Neuropharmacology.

[15] Carvajal-Gonzalez A, Leite MI, Walters P (2014). Glycine receptor antibodies in PERM and related syndromes: characteristics, clinical features and outcomes. Brain.

[16] Ken-Ichi I, Takahisa T, Taiga M (2022). Anti-glycine receptor antibody-positive progressive encephalomyelitis with rigidity and myoclonus initially presenting with one-sided stiff face: a case report. Front Neurol.

[17] Crisp SJ, Dixon CL, Jacobson L (2019). Glycine receptor autoantibodies disrupt inhibitory neurotransmission. Brain.

